# The Alkalinity of the Blood in Disease

**Published:** 1908-08-01

**Authors:** 


### Pathology.

### THE ALKALINITY OF THE BLOOD IN DISEASE.

A good deal of literature exists upon the subject of the alkalinity of the blood in health and in disease, and many observers have expressed the opinion that valuable clinical information can be obtained from the estimation of this alkalinity in different cases. It is always of importance to know what laboratory methods are helpful in forming a more accurate opinion as to diagnosis or prognosis; it is equally of importance to be able to relegate unhelpful clinical methods to their proper place —possibly with some sense of relief. Some extensive researches carried out by Dr. Solomon Strouse in the Clinical Laboratory at the Johns Hopkins Hospital show that the question of variations in the alkalinity of the blood is not at present one of practical interest to those engaged in seeing and treating patients, whatever the purely scientific interest of the question may be. Dr. Strouse says in his summary: "Remembering the limitations of clinical methods we should be satisfied if the results of any such method of hæmalkalimetry had aided the internist in diagnosis, prognosis, or therapy; but so far all methods have been devoid of even empirical value. Further, until the time comes when we have a definite knowledge of the causes underlying relatively slight variations in the neutralisable alkali we can hope for little of practical interest from these observations."

Beyond merely stating the broad fact in this way, it may be well to indicate some of the reasons why hæmalkalimetry is so difficult as to be of little or no practical utility.

The blood is not an alkaline fluid in the sense that a solution of sodium hydrate is alkaline. The same applies equally to urine, the alkalinity or acidity of which is nearly as complex and as difficult to estimate. Blood is a very complex mixture of salts, mainly carbonates and phosphates of the alkaline bases, together with other substances, such as proteins, which may combine with either alkalies or acids. Probably its most important constituents from the point of view of alkalinity are sodium bicarbonate and sodium phosphate, which are really acid salts with an alkaline reaction to many reagents. As a matter of fact, all recent studies in physical chemistry have proved the practical neutrality of the blood, while Friedenthal by most interesting biological deductions has arrived at the same conclusion.

Moreover, it is extremely difficult to make the blood either more or less alkaline by any experimental measures such as the direct transfusion of weak solutions of hydrochloric acid into the circulatory system of animals. The body has a very facile method of compensating for any diminished alkalinity that might be expected from such proceedings, in that the urea which appears in the urine is synthesised by the liver from ammonium carbonate, and as soon as there is any excess of acid introduced into the system some of the ammonium carbonate is immediately utilised to combine with and neutralise it instead of being converted into urea. The result is that the alkalinity of the blood remains the same, but the amount of ammonium salts in the urine increases and that of urea falls. Herbivora have this power of compensation in very much less degree than have carnivora; man seems to occupy an intermediate position. More is to be expected from analysis of the urine for combined ammonia than from the very difficult estimations of the alkalinity of the blood.

It can be shown by simple titrations that the alkaline reaction of blood is not like that of a solution of sodium hydrate. Blood will give varying figures of alkalinity when different indicators are used. To phenol-phthaleïn it is practically neutral; to litmus its alkalinity is represented by about 50 milligrams of sodium hydrate per 100 cubic cm. of serum; to di-methyl-amido-azo-benzol by about 300 milligrams of sodium hydrate per 100 cub. cm. Now 100 cubic cm. of a decinormal solution of sodium hydrate will be neutralised by very nearly 100 cubic cm. of decinormal sulphuric acid, no matter what indicator is used; but the amount of decinormal sulphuric acid required to neutralise 100 cubic cm. of decinormal sodium carbonate, sodium bicarbonate, or di-sodium phosphate, varies greatly with the indicator used; in this respect the reaction of the three latter salts is precisely analogous to that of the blood.

Clinical methods are not necessarily as accurate as chemical laboratory methods, and at best they are often a compromise between accuracy and simplicity. They must be measured by the results obtained; if they are only of comparative value and yet of service clinically, they can be considered useful. So far, however, notwithstanding the fact that over thirty-five different methods have been devised for getting over the various difficulties that arise, there is as yet no method of hæmalkalimetry that is of even comparative clinical value. The results obtained depend to a great extent on the nature of the acid used and of the indicator employed. Further, some difference in results will be observed, depending on whether one titrates to neutrality, or whether one adds an excess of acid and then titrates the uncombined acid with alkali. In the first case one measures simply the neutralisable alkali, in the second one is dealing with a more complex question owing to the presence of proteins, urea, and other bodies which may take up the excess of acid and yet do not influence the indicator. This means that the total basicity and the neutralisable alkali of the blood are not the same.

The values for normal have varied, in the hands of different observers, using different methods, from 182 milligrams of sodium hydrate per 100 cubic cm. of blood to 533 milligrams. When the variations for healthy blood are so great, it is not surprising that the determinations in disease do not give figures which lie sufficiently outside the normal limits to be of clinical value. That is the conclusion at which Dr. Strouse arrives after the investigation of a large number of cases of various diseases.

